# The Role of Organoids as a Novel Platform for Modeling of Inflammatory Bowel Disease

**DOI:** 10.3389/fped.2021.624045

**Published:** 2021-02-17

**Authors:** Lauren O'Connell, Des C. Winter, Carol M. Aherne

**Affiliations:** ^1^Conway Institute of Biomolecular and Biomedical Research, School of Medicine, University College Dublin, Dublin, Ireland; ^2^Centre for Colorectal Disease, St. Vincents' University Hospital, Dublin, Ireland

**Keywords:** organoids, inflammatory bowel disease, disease modeling, mucosal defense, epithelial barrier

## Abstract

Inflammatory bowel disease (IBD) is a chronic relapsing-remitting immune-mediated disorder affecting the gut. It is common in Westernized regions and is increasing in incidence in developing countries. At a molecular level, intrinsic deficiencies in epithelial integrity, mucosal barrier function, and mechanisms of immune response and resolution contribute to the development of IBD. Traditionally two platforms have been utilized for disease modeling of IBD; *in-vitro* monolayer cell culture and *in-vivo* animal models. Both models have limitations, including cost, lack of representative cell types, lack of complexity of cellular interactions in a living organism, and xenogeneity. Organoids, three-dimensional cellular structures which recapitulate the basic architecture and functional processes of the organ of origin, hold potential as a third platform with which to investigate the pathogenesis and molecular defects which give rise to IBD. Organoids retain the genetic and transcriptomic profile of the tissue of origin over time and unlike monolayer cell culture can be induced to differentiate into most adult intestinal cell types. They may be used to model intestinal host-microbe interactions occurring at the mucosal barrier, are amenable to genetic manipulation and can be co-cultured with other cell lines of interest. Bioengineering approaches may be applied to render a more faithful representation of the intestinal epithelial niche. In this review, we outline the concept of intestinal organoids, discuss the advantages and disadvantages of the platform comparative to alternative models, and describe the translational applications of organoids in IBD.

## Introduction

Inflammatory bowel disease (IBD) is an immune-mediated relapsing-remitting chronic disorder affecting the gut. Alterations in the intestinal microbiome, defects in mucosal barrier defense and aberrant innate and adaptive immune responses appear to be critical to the development of IBD (1–7). Clinically, two major phenotypes exist, Crohn's disease (CD) and ulcerative colitis (UC). CD features transmural inflammation in a “skip lesion” or discontinuous pattern. Although it may affect any part of the gut the terminal ileum is most frequently involved (1, 2, 8–10). UC affects the colon only, although a reactive “backwash ileitis” may occur. Inflammation is limited to the mucosa and submucosa and occurs in a continuous pattern, with rectal involvement extending proximally for a variable distance. Crypt abscesses due to accumulation of neutrophils are characteristic (5, 6, 11).

## Pathogenesis

The underlying mechanisms which contribute to the etiology of IBD are highly complex and not yet fully elucidated. Genetic susceptibility, environmental factors, defects in mucosal barrier function, immune dysregulation, and dysbiosis have all been demonstrated to contribute to disease pathogenesis (12–18). Activation and recruitment of CD4+ T cells to the intestinal tissue and production of a proinflammatory cytokine cascade, particularly the Th1- and Th17-associated cytokines TNFα, IFNγ, IL-12, IL-21, and IL-23 in CD and Th2-associated cytokines IL-4 and IL-13 in UC are commonly observed (5, 19–21).

Loss of a functional epithelial barrier and increased permeability of the mucus gel layer, permits abnormal contact of luminal organisms with the epithelium, provoking an inflammatory response from the immune system located in the lamina propria (14, 22–25). Failure of inflammation to resolve along with lack of restoration of normal mucosal homeostasis results in progression to chronic inflammation, inadequate epithelial restitution, and ongoing tissue damage (26). This is accompanied by characteristic disturbances in the composition of the gut microbiome, with a reduction of obligate anaerobes such as Firmicutes, an increase in facultative anaerobes such as Enterobacteriaceae and the presence of invasive strains such as adherent-invasive *E.coli* (AIEC) (27–30).

Genome-wide association studies have thus far identified up to 250 susceptibility loci involved in IBD. The most well-known of these is the Crohn's susceptibility locus CARD15, formerly known as NOD2, which is responsible for sensing of luminal bacterial organisms; others include *IL23R* and *ATG16L1*, which play roles in IL23 signaling and autophagy, respectively (13, 31, 32). Many susceptibility loci are genes coding for components of the mucosal barrier. These include proteins responsible for assembly and maintenance of epithelial tight junctions, intercellular adhesion and polarity, mucin and glycoprotein synthesis, bacterial sensing mechanisms, and epithelial wound healing and restitution (13, 33, 34).

Current therapeutic strategies in IBD primarily function by modification of the immune response. Biologic therapies targeting the cytokines TNFα, IL-12 and IL-23, and integrin blockers which limit the migration of leukocytes to the GI tract have greatly expanded the repertoire of treatment options (35–37). However, up to 40% of patients fail to respond to biologic therapies, and up to 50% develop secondary treatment failure after an initial successful response (38). Although impaired barrier function is also a critical event in initiation and perpetuation of IBD no therapies directed at augmenting the barrier deficiency which occurs in IBD have successfully been developed for clinical practice. Due to the phenomenon of treatment-resistant IBD in a substantial proportion of patients, alternative strategies aimed at improving intestinal barrier function are warranted. Development of such therapies requires highly faithful modeling of the intestinal barrier in the preclinical setting.

## Current Models of IBD

Traditional models for IBD comprise animal models and monolayer cell culture. Some animal models used to study IBD such as DSS-colitis and TNSB-colitis are extensively utilized and well-described (39, 40). In addition to chemically induced colitis, the creation of transgenic and knockout animal strains permit investigation of inflammation arising from specific defects in innate and adaptive immune responses (41–44). These models have the benefit of replicating the complex organization and simultaneous interactions that occur in the gut in a whole organism. Such models have been indispensable in unraveling the complex pathophysiology and molecular abnormalities that occur in IBD.

However, *in-vivo* disease modeling in animals does have some limitations. Chemical induction of colitis occurs by a heterogeneous mechanism to that by which inflammation occurs in human disease. While cell culture can be rapidly established, the length of animal reproductive cycles means that animal experiments are a slower process. Ethical considerations exist with the use of higher vertebrates which do not apply to cell culture. In addition, while the host-microbial interactions and inflammatory processes that occur in animal models are broadly applicable to humans, particular aspects of the microbiome, inflammatory response, and mucosal defense may be species-specific (45–47). Finally, animal models are poorly predictive of drug response and toxicity in humans (48, 49).

*In-vitro* immortalized intestinal human cell lines such as Caco-2, T84, and HT-29 cultures are excellent for investigating specific molecular interactions and signaling pathways under highly controlled conditions. They are derived from human tissue, are low-cost and can be rapidly established. However, monolayer cultures are reductive as a model and cannot replicate the complex interactions that occur *in-vivo*.

## Organoids

Organoids are defined as 3-D structures derived from either pluripotent (embryonic or induced pluripotent), or adult tissue-resident stem cells, which spontaneously self-organize and undergo a degree of differentiation, producing functional cell types, and which have the capacity to undertake some functions of the relevant organ (50).

While systems for maintaining intestinal tissue explants *ex-vivo* had been described since 1992, (51, 52) it was Eiraku et al. (53) and Sato et al. (54), respectively, who first successfully developed a method of producing the stem-cell derived, constructs known today as organoids. Studies by Sato et al. derived these from Lgr5+ adult stem cells (ASCs), first from murine and subsequently human intestinal crypts. They self-organized into crypt-villus type architecture and had the potential to produce most mature cell lines of the gut (54–58). Since then organoid cultures have been successfully derived from other anatomical locations, including colonic, gastric and esophageal tissue (55, 56, 59–62).

Organoids derived from small bowel tissue are sometimes referred to as enteroids or simply small bowel organoids, while organoids derived from colonic tissue may similarly be referred to as colonoids. They can be expanded from small volumes of tissue, including from endoscopic biopsies. Lgr5^+^ ASCs can be induced to differentiate into organoids containing all cell lines propagated by the gut, including mature enterocytes, Paneth cells, goblet cells, enteroendocrine, and tuft cells (48, 54, 55, 63). PSC-derived organoids can additionally generate adjacent stromal cell types. They recapitulate the spatial organization and polarity observed in the intestinal mucosa. Gut organoids are also capable of many of the functions of the source tissue, including endocrine and paracrine secretion, filtration, molecular transport, absorption, and contraction (48).

By contrast, while cheap and rapidly established, immortalized monolayer cell lines cannot recapitulate the complex cell-cell interactions or interactions with the extracellular microenvironment which occur in whole organisms (64, 65). Typically only single cell types are represented (66). It is not possible to culture rarer intestinal cell types such as tuft cells, and it can be difficult to acquire immortalized cell lines which secrete mucus to mimic the mucosal barrier which exists *in-vivo* (48, 61). Further, as monolayer cell cultures are derived from malignant cells they intrinsically demonstrate different properties to those of non-malignant cells, particularly with respect to epithelial integrity, cell polarity, and adhesion. These cells are not fully differentiated, and cell division in monolayer cell culture does not respond to the usual cellular signaling mechanisms which regulate this process *in-vivo* (48, 61, 67) (Table 1).

**Table 1:** Characteristics of different modeling platforms in IBD.

**Feature**	**2D cell culture**	**Animal models**	**Organoids**
Cost	+	++	++
Culture cycle length	+	+++	++
Presence of all intestinal cell types	–	++	++
Presence of non-epithelial elements of intestinal niche	–	+++	+/–
Genetic stability	+	+++	++
Suitability for high-throughput studies	++	–	++
Suitability for drug toxicity screening	–	++	++

Organoids theoretically have the potential to bridge the gap between monolayer cell culture and whole-organism environments. They are derived from human tissue and recapitulate the complex cellular organization seen *in-vivo*. However, they avoid the issues of xenogeneity which may be associated with animal models (68, 69). Organoids are also less costly and can be more rapidly established than animal models while retaining the potential for highly controlled molecular and genetic manipulation which is the salient attractive feature of monolayer cell culture (Table 1).

## Translational Application of Organoid Models in IBD

### Physiological Modeling of the Intestinal Niche

Differential protein expression, gene expression, cell migration, organization, survival, and cell signaling have been observed in organoid cultures comparative to monolayer cell culture (70–73). Defects in the function of multiple epithelial cell types have been demonstrated in IBD, underlining the need for a physiologically relevant model which includes multiple cell lineages (74–78). Gut organoids may also be co-cultured with non-epithelial cell lines of interest in order to more accurately represent the intestinal mucosal niche. Co-culture of gut epithelial organoids with cell lines such as macrophages and lymphocytes and with mesenchymal cells demonstrate promise in providing a more physiologically relevant model of the gastrointestinal mucosal environment (79–82).

### Host-Microbe Interactions

Due to this ability to accurately simulate the intestinal microenvironment, intestinal organoids represent exciting models for investigating the host-microbial interactions which are key to the pathogenesis of IBD. Organoids have already been successfully utilized as a more accurate model for human virus infection. In a study by Saxena, fully differentiated cells present in organoid culture supported greater rotavirus viral load and replication than had been previously observed in monolayer culture; and infection of enteroendocrine cell types in addition to enterocytes with rotavirus was demonstrated (83). Organoids have also been used to investigate norovirus, which is difficult to cultivate in monolayer cell culture. Previously only successfully cultured in B cells, organoids permitted culture of norovirus in duodenal, jejunal, and ileal cell types with viral replication and growth occurring within (84). Current applications of organoids include disease modeling of SARS-CoV-2 in respiratory and small intestinal derived cell types, with viral infection, replication, and host viral response observed *ex-vivo* (85–87).

Organoid cultures have also been applied to simulate host-bacterial interactions. Salmonella, *H. pylori, C. difficile*, and pathogenic *E. col*i infection have all been modeled in organoid cultures (66, 88–91). In one study, gastric organoids which secrete mucous, include multiple epithelial cell types and retain the polarity of the *in-vivo* gastric epithelium have been successfully utilized as a model for host-microbe interactions in *H.pylori* infection (66). Interestingly, duodenal, ileal and colonic organoid cultures derived from different donors demonstrate a differential response to infection and differing patterns of bacterial adhesion, possibly due to the genetic variability based on the tissue of origin (89). A co-culture model developed to study the host-pathogen interactions of *C. jejuni* incorporates intestinal enterocytes, mucin-secreting goblet cells and dendritic cells, thus combining a mucus-secreting epithelial layer with cellular elements of the intestinal innate immune system (92).

**Figure 1:**
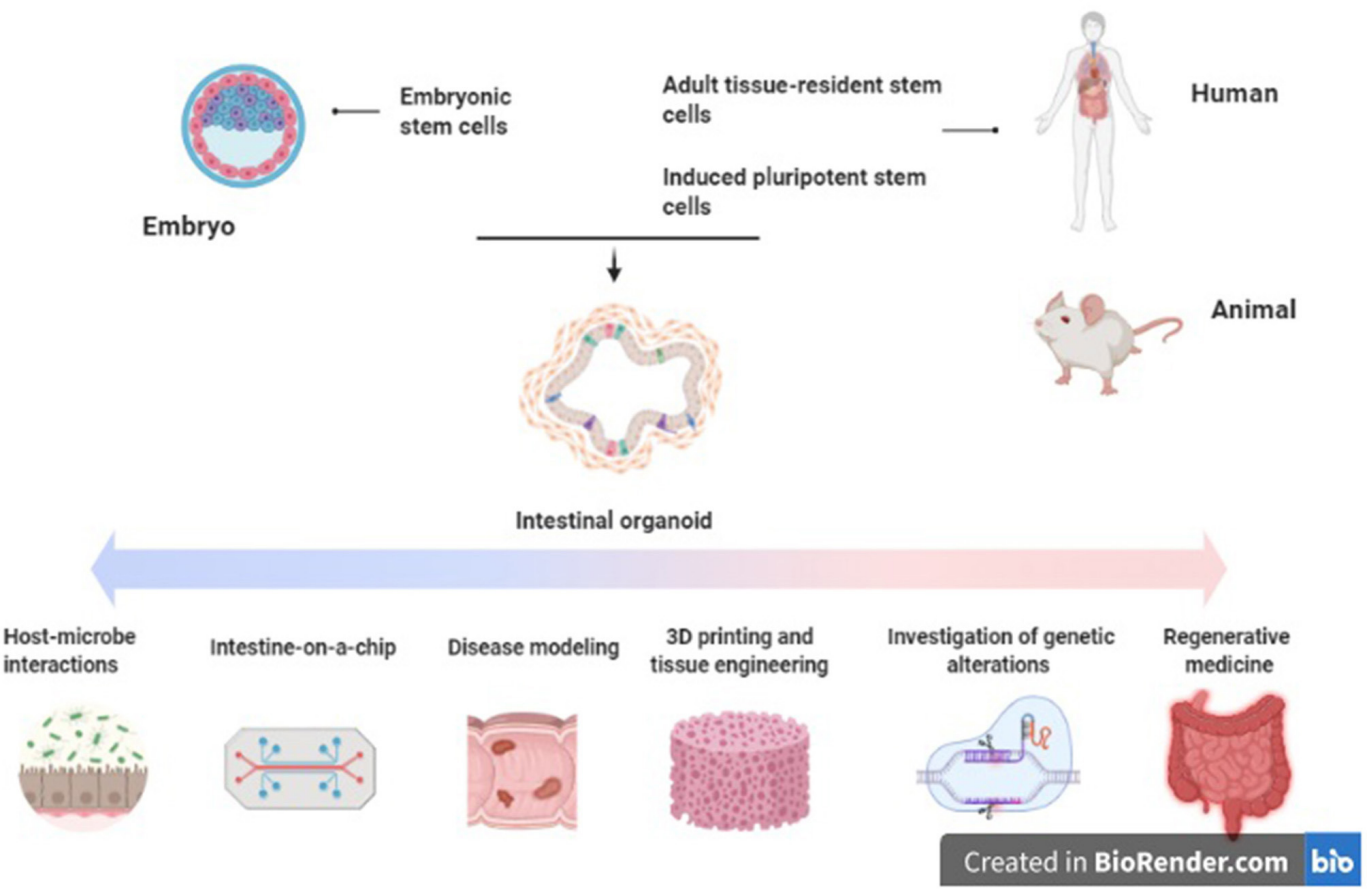
Applications of intestinal organoids in inflammatory bowel disease. Original figure (created with BioRender.com).

As well as modeling invasive microorganisms, organoids can also be used to study interactions between the gut and commensal microbiota. In one study, microbiota were found to play a role in epithelial regeneration in murine small bowel organoids. The pattern recognition receptor NOD2, single nucleotide polymorphisms (SNPs) of which are highly associated with Crohn's disease, is highly expressed in mouse intestinal stem cells (93–96). Stimulation of NOD2 by MDP (peptidoglycan muramyl-dipeptide), a bacterial cell wall constituent, enhanced organoid survival and protected them from oxidative-stress mediated cell death (96, 97). Organoids derived from adult and fetal murine tissues have also been utilized to determine developmental expression patterns of components of the innate immune system, including NOD2, TLR4, and TLR5 (98). Exposure of murine intestinal organoids to gut commensal bacteria including *Akkermansia muciniphilia* and *Faecalibacterium prausnitzii* has been shown to induce changes in gene expression and transcription, particularly of genes responsible for lipid metabolism (99). Similarly, exposure to the organism *Bacteroides thetaiotaomicron* and cytokine signaling via IL-22RA1 induces upregulation of Fut2 and increased fucosylation, which in turn inhibits colonization by opportunistic *Enterococcus faecalis* strains (97, 100, 101). Finally, alterations in the microbiome have been associated with colonic neoplasia; colonic organoid models have been used to demonstrate a mutational profile induced by exposure to colibactin synthesized by genotoxic *E. coli* which is also associated with colorectal cancer *in-vivo* (102). Thus, organoid systems may be utilized to explore activity of the gut microbiome on the epithelium and mechanisms of homeostasis (Figure 1).

### Disease Modeling

Unlike organoids derived from pluripotent stem cells, which rapidly accumulate mutations and epigenetic modifications, ASC-derived organoids are relatively genetically stable (103, 104). They retain the genetic profile and also the transcriptional and epigenetic landscape of the primary tissue from which they are derived (73, 105–107). While the majority of IBD is polygenic, some monogenic forms exist. These are mediated by specific genetic defects in epithelial dysfunction and stress response, defects in immune regulation of regulatory T cells or immunodeficiencies of phagocytic cells (108). Organoids represent useful models for studying these rare diseases, as well as other genetically determined intestinal disorders (109).

It is possible to culture intestinal organoids derived from patients with active IBD (110, 111). In one study, IBD colonic organoids demonstrated a distinct phenotype to those derived from control tissue, with a smaller size, increased cell death, abnormal cell polarization, and poorer budding capacity (110). Interestingly, they also expressed reduced quantities of the tight junction proteins ZO1, Occludin, and Claudin-1 as well as alterations in the expression of adherens junction and desmosomal proteins. These altered expression patterns persisted when the inflammatory stimulus was withdrawn (111). The phenotype and altered transcriptional profile noted in the IBD-derived organoids was inducible in the control organoids with administration of pro-inflammatory cytokines (TNFα, IL-1, and IL-6). Hibiya et al. demonstrated that murine colonic organoids which are exposed to chronic inflammatory stimuli (TNFα, IL-1β, IL-6, LPS, flagellin) underwent upregulation of the NFκB signaling pathway, which persisted after stimuli removal (112). These organoids also underwent transformation to an undifferentiated state, along with upregulation of genes related to oxidative stress and carcinogenesis (Smox and CD151), suggesting their potential utility as a model to study the epithelial changes which occur in colitis-induced carcinogenesis. Vermeire et al. also generated CD and UC-derived organoids which were subsequently exposed to TNF and flagellin, resulting in modulation of expression of the SARS-CoV-2 receptor ACE2. These changes were restored to baseline with anti-TNF treatments (113). Other studies utilizing patient-derived organoids from pediatric IBD patients demonstrated alterations in DNA methylation and transcriptional profiles, which correlated with treatment outcomes (114). Finally, a study by Jardine et al. successfully used colonic organoids generated from patients with TTC7A deficiency to perform high-throughput drug screening for candidate therapeutic agents (115). Loss of TTC7A causes intestinal epithelial apoptosis and immune defects which presents clinically as very early onset IBD. Thus, primary organoid cultures from inflamed tissue seem to represent an applicable model for investigation of the epithelial and mucosal abnormalities which occur in IBD (Figure 1).

### Bioengineering and Gut-on-a-Chip

In the small intestine the mucosa of the gut is folded into villi and microvilli to maximize available surface area for absorption. Bioengineering techniques such as 3D printing and laser ablation allow the creation of scaffolds which recreate this intestinal topography. These can be directly seeded with epithelial organoids or used as molds to create hydrogel-based porous copies which reproduce the microanatomy of the gut (116). Alternatively, bioink comprising cell aggregates or organoids may be imprinted along with the desired biomaterials (hydrogels, matrix components) onto the scaffold via a computer-aided transfer process (117, 118). This allows for the creation of highly accurate and reproducible models with each component—organoids, biomaterial, and scaffold—spatially aligned at the desired patterns, gradients, and densities set by the modeling software. Such methods will help to address both reproducibility and scaling-up of organoid cultures into larger tissue constructs. Some 3D gut models aimed at investigating the pathophysiology of inflammatory bowel disease are already in use (119).

Aside from gut anatomy, intestinal motility and luminal flow are physiologic functions of the gut which are difficult to mimic *ex-vivo*. These can be simulated via epithelial cell-lined microfluidic platforms, sometimes referred to as a “gut-on-a-chip” (120–122). Such platforms permit recapitulation of flow patterns, mechanical deformation, shear stresses, and peristaltic activity with greater accuracy than has been possible previously (117, 123–125). Organoid-lined laser-ablated microchips with active perfusion of media components have also been developed, which permit simulation of intestinal homeostasis and cell turnover with a reduced need for passaging (126). These platforms are being utilized to further investigate the gut-microbiome relationship by inoculation with bacterial cultures and examining the effect of the physical environment on intestinal host-microbe interactions (127–132) (Figure 1).

### Regenerative Medicine

The concept of mucosal healing as key to sustained remission of IBD has become increasingly prominent in recent years. This denotes absence of all mucosal ulceration at endoscopy, rather than resolution of clinical symptoms and serum biomarkers of inflammation alone (133, 134) Mucosal healing correlates with improved long-term clinical outcomes, including steroid use, hospital admissions and need for surgery in both CD and UC (135–139). The European Crohn's and Colitis Organization lists mucosal healing as a therapeutic target in its 2017 consensus guidelines for both UC and CD (140, 141). Local transplant of organoids to aid mucosal healing has been proposed as a potential therapy in IBD to aid epithelial regeneration and achieve mucosal healing (142). Studies using murine colitis models have demonstrated that human small bowel and colonic organoid cultures can engraft onto the ulcerated mucosa and reconstitute the normal crypt-villus architecture (58, 143, 144). More recently, patient-derived small intestinal organoids have been successfully expanded *ex-vivo* and engrafted into mice, with the ultimate aim of creating autologous small intestinal transplants to treat intestinal failure (145) (Figure 1).

### Limitations of Organoids as a Model Platform

Despite the advantages described above, there are limitations associated with the use of organoids. Comparative to two-dimensional models they are more costly and less accessible, and require specialized medium to be maintained in culture. Matrigel and similar matrices in which they are typically cultured are expensive and increase the difficulty of manipulation. Particular studies such as transport and luminal exposure studies require injection of organoids which is a technically difficult and labor-intensive procedure; alternatives such as computer-assisted injection are again expensive and not readily available. Access to human tissue for generation of primary organoid cultures can be limited (78, 146). They are typically derived from epithelial tissues and so other components of the intestinal niche, including immune and mesenchymal elements, are underrepresented (146). As they are three-dimensional structures, this presents difficulty for investigations requiring access to the apical and basolateral surfaces. For this purpose they may be dissociated into 2D structures; however this disrupts their crypt-villus architecture and terminates their culture cycle (146). Finally, reproducibility of organoid cultures is challenging, as constructs of differing sizes and morphology result when they are grown *in-vitro*.

## Conclusion

In summary, intestinal organoids represent a promising novel platform for further elucidating the host-microbe interactions, mucosal barrier deficiencies and genetic defects which underpin the pathogenesis of inflammatory bowel disease. Patient-derived organoids may have translational applications in the future as local therapy to aid mucosal healing. However, many limitations yet remain with this model. Some of these may be addressed by innovations such as computer-assisted bioprinting and 3D printed scaffolds to aid in reproducibility, and development of co-culture systems including immune and neuronal components to increase the physiological relevance of organoids as a platform for investigation of IBD.

## Author Contributions

LO'C: preparation of manuscript. DCW and CMA: concept, review and editing of manuscript, and approval prior to submission. All authors contributed to the article and approved the submitted version.

## Conflict of Interest

The authors declare that the research was conducted in the absence of any commercial or financial relationships that could be construed as a potential conflict of interest.
